# Unlocking the Nutritional Potential of Gamma Amino Butyric Acid (GABA) Rice: Towards a Sustainable Approach to Metabolic Disorder Remedies

**DOI:** 10.1002/fsn3.71284

**Published:** 2026-02-17

**Authors:** Uloma E. Onyeka, Ekpeno Sunday Ukpong, Chinedu B. Azudialu, Sali A. Ndindeng, Kalimuthu Senthilkumar, Christian O. Dimkpa

**Affiliations:** ^1^ Department of Food Science and Technology, School of Engineering and Engineering Technology Federal University of Technology Owerri Imo Nigeria; ^2^ Department of Human Nutrition and Dietetics Alex Ekwueme Federal University Ndufu‐Alike Ebonyi Nigeria; ^3^ Federal Medical Centre Owerri Imo Nigeria; ^4^ Africa Rice Center (AfricaRice) Bouake Côte d'Ivoire; ^5^ Africa Rice Center (AfricaRice) Antananarivo Madagascar; ^6^ Connecticut Agricultural Experiment Station New Haven Connecticut USA

**Keywords:** gamma amino butyric acid, germinated brown rice, paddy rice, phytic acid

## Abstract

This study explores the nutritional enhancement of germinated brown rice (GBR), commonly referred to as GABA rice, produced from Nigerian rice cultivars (FARO 44, FARO 52, and FARO 57). Paddy rice was germinated at 35°C for 0 (control), 12, 24, and 36 h, followed by parboiling and dehusking to obtain GBR. Nutritional analyses focused on mineral, vitamin, and phytic acid contents. Germination significantly increased macro‐minerals, including calcium, magnesium, phosphorus, and sodium, by up to 109.8%, 22.1%, 12.1%, and 149.0%, respectively after 36 h of germination. The levels of trace minerals such as zinc, iron, manganese, selenium, and cerium also increased, while phytic acid level decreased with longer germination durations. Vitamin content improved markedly, with increases of 387.5% for B1, 440.0% for B2, and 56.8% for vitamin E, after 36 h of germination. A 24 h germination period was optimal for mineral enrichment, while 36 h maximized the vitamin levels. These findings suggest that GBR, when integrated into conventional rice processing systems, could offer a cost‐effective dietary intervention for addressing micronutrient deficiencies and managing metabolic health disorders.

## Graphical abstract

This study addresses the critical issue of nutrient deficiencies associated with the widespread consumption of conventional milled rice, which often lacks essential vitamins and minerals due to the removal of the bran layer during processing. It demonstrated that controlled germination of paddy rice, followed by parboiling, substantially increases the levels of essential minerals such as calcium, magnesium, phosphorus, and iron, as well as vital vitamins including B1, B2, B3, B6, and E, while lowering the phytic acid levels. Additionally, the study provides insights into the impact of different rice cultivars and germination durations on nutrient enhancement, contributing to the growing body of evidence supporting the use of germination as a natural biofortification method, and offering a sustainable and effective strategy to improve the nutritional profile of rice.

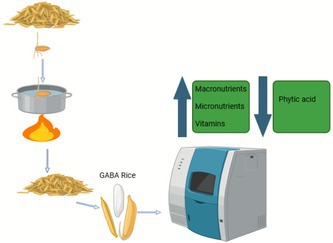

## 1 Introduction

Rice ( *Oryza sativa* L.) is a staple crop that feeds nearly half of the global population. The grain is harvested as paddy, which consists of a protective husk enclosing the bran, germ, and starchy endosperm (Yu et al. 2024; Awio et al. 2021). Milling typically removes the husk and bran, resulting in white rice with significantly reduced levels of lipids, vitamins, minerals, and phytochemicals (Sathe et al. 2020; Ukpong et al. 2023). Although brown rice retains the nutrient‐rich bran layer, its adoption by consumers is limited due to its chewy texture, long cooking time, and susceptibility to rancidity (Maksup et al. 2021; Ren et al. 2020).

Germination has been shown to improve both the nutritional profile and palatability of brown rice. The process of germination activates hydrolytic enzymes that soften the grain, enhance aroma, and increase the concentrations of health‐promoting compounds such as gamma‐aminobutyric acid (GABA), polyphenols, and antioxidants (Duenas et al. 2009). In a previous study, we germinated rice under controlled conditions and measured the gamma‐aminobutyric acid content, which showed significant increase as a function of germination duration (Ukpong et al. 2023); hence, the name GABA rice. Germination causes the endogenous dormant enzymes in the rice bran layer to degrade the starch, protein and lipid to release the bound materials and generate different beneficial bioactive compounds including GABA (Ukpong and Onyeka 2019; Ukpong et al. 2024). The high amylase activity during germination breaks down starch and hence, the reduction in starch contents and increase in reducing sugar of GBR. Germination also results in the development of new cell walls which increased dietary fiber contents (do Nascimento et al. 2020). It also lowers the levels of antinutritional factors such as phytate, resulting in increased bioavailability of the dietary minerals (Chinma et al. 2015; Sathe et al. 2020; Zhu et al. 2024). GABA is a non‐protein amino acid that serves as a neurotransmitter and is associated with reduced blood pressure, cholesterol, and oxidative stress, as well as improved glucose regulation and neuroprotection (Zhao et al. 2018; Weng et al. 2019). GABA is reported to improve the overall metabolic activities in diabetics, achieved through the stimulation of insulin‐like growth factor‐1 and reduction in oxidative stress, which is a problem among type‐2 diabetic patients (Binh et al. 2020; Nguyen et al. 2021; Zhu et al. 2024). Moreover, food rich in GABA, when consumed, lowered blood pressure and cholesterol levels in human and animal subjects (Nguyen et al. 2021; Weng et al. 2019; Zhao et al. 2018).

FARO 44, FARO 52, and FARO 57 rice cultivars are among the cultivars recommended by the Africa Rice Center (AfricaRice) and are widely cultivated by farmers in Nigeria due to their desirable characteristics. Faro 44 is a medium‐to‐tall variety adapted to lowlands, with an early maturity period of 90–110 days, and a potential to yield up to 4–5 tons per ha of long‐grain seeds. It possesses moderate tolerance to common foliar diseases. FARO 52 is also a lowland variety with a maturity period of 123–135 days. It is tolerant to iron toxicity and drought, conferring it with a high yield propensity of 5–6 tons per ha, even under limited water conditions. FARO 57 is also lowland‐adapted, with a medium maturity of 100–120 days. It is resistant to a variety of abiotic and biotic stresses, including drought, iron toxicity, blast, and rice yellow mottle virus disease, and permitting a potential yield of 6–9 tons per ha of long slender grains (Oluwaseyi et al. 2016).

Information is available on the nutritional composition of GBR of other rice cultivars grown in other countries (e.g., Lee et al., 2019; Zhu et al. 2024). However, information on the physico‐chemical properties, nutritional content and bioactive compounds of three of the most popular rice cultivars in Nigeria, namely FARO 44, FARO 52, and FARO 57 is limited. Notably, the few available studies focused on germinating dehusked brown rice, a process that can lead to grain breakage and nutrient loss through leaching or fermentation (Ukpong et al. 2023, 2024). The present study, instead, explores germination of *paddy* rice prior to parboiling and dehusking as a method that aligns with existing industrial practices and may yield greater nutrient retention. Parboiling after germination can promote the migration of nutrients into the endosperm and reduce nutrient loss during the milling process (Kumar et al. 2018; Muchlisyiyah et al. 2023).

The broader objective of this study is to evaluate the effects of controlled germination (0–36 h) on the nutritional and physico‐chemical (including minerals, vitamins, phytic acid, and proximate, among others) qualities of GBR derived from rice cultivars FARO 44, FARO 52, and FARO 57. The specific aim is to identify optimal germination durations, in combination with parboiling, that maximize the nutritional value and support the use of GBR as a functional food for combating micronutrient deficiencies and chronic metabolic diseases (Bhullar and Gruissem 2013; Birla et al. 2017).

## 2 Materials and Methods

### 2.1 Source of the Rice Paddy

Paddy rice of three popular cultivars grown in Nigeria, namely ARO 44, FARO 52, and FARO 57, was procured from a local farm in Abakaliki, Ebonyi State, southeastern Nigeria. As noted above, these cultivars are widely cultivated and recommended by the Africa Rice Center (AfricaRice) due to their desirable agronomic qualities. These cultivars have high consumer demand, and the grains have medium height which resist heavy wind giving high yield of 4.5–4.7 tons per hectare.

### 2.2 Production of Germinated Brown Rice

The method described by Ukpong et al. (2022) was adapted with modifications. Instead of dehusked brown rice, intact paddy was used for germination. Paddy (25 kg) was sterilized by soaking in 0.1% sodium hypochlorite solution for 30 min, followed by thorough rinsing with potable water to remove excess Na. It was then steeped in potable water (ratio of 1:3 paddy: water) at room temperature (30°C ± 2°C) for 12 h, with water changes every 6 h to prevent fermentation. After soaking, the rice was spread on moistened jute bags placed on stainless steel trays, covered with an additional moistened jute bag, and incubated at 35°C. Samples were harvested at 0 (control), 12, 24, and 36 h, representing different germination durations. Each batch was steamed at atmospheric pressure for 10 min, oven‐dried at 120°C for 10 min, then further dried at 78°C until moisture content was below 13%. Finally, the samples were dehusked using a SATAKE rice husker (Australia) to obtain the GBR.

### 2.3 Determination of Proximate Composition and Functional Properties of GBR Samples

The samples were first milled to flour using a hammer mill. The nitrogen value (N) by Kjeldahl, the crude protein (N × 6.25), fat (solvent extraction with petroleum ether), total dietary fiber (enzymatic‐gravimetric method), ash, total carbohydrate (phenol and sulfuric acid method) and moisture contents were determined by AOAC (2015) methods. Functional parameters including bulk density, water absorption capacity, oil absorption capacity, foaming capacity, foam stability and swelling power were determined on the different flour samples by AOAC (2015). Fifty grams of flour was measured into a 100 mL graduated measuring cylinder. This was followed by tapping the bottom of the cylinder on the laboratory table until there was no further change in volume after which the volume was recorded. The bulk density (gml^−1^) was then calculated using Equation (1).

(1) $$\mathrm{BD}=\frac{\mathrm{WF}}{\mathrm{VF}}$$

where BD = bulk density (gmL^−l^); WF = weight of flour (g); VF = volume of flour (mL).

For oil absorption capacity 1 g of the sample was weighed into a graduated conical flask and 10 mL of distilled water was added. This was followed by whirling for 30 s to mix. The sample was allowed to stand at room temperature (29°C ± 2°C) for 30 min after which it was centrifuged at 5000 rpm for 30 min. The mixed sample was finally poured into a 10 mL measuring cylinder to determine the volume of the free water. The water absorption capacity was calculated using Equation (2).

(2) $$\mathrm{WAC}=\left(\mathrm{TWA}-\mathrm{FW}\right)\times \mathrm{DW}$$

where WAC = water absorption capacity; TWA = total water absorbed; FW = free water (supernatant); DW = density of water.

### 2.4 Determination of Total Starch, Amylose and Reducing Sugar Compositions and Structure

The method described by AOAC (2015) was used to determine the percentage of total starch and reducing sugar in the samples while ISO (2015) was used for amylose content determination. For starch, the sample was treated with ‐amylase and amyloglucosidase before diluting with acetate buffer and addition glucose oxidase/peroxidase reagent and the absorbance was read against the blank at 510 nm wavelength. The total reducing sugar was determined by titrating the sample extract with 20 mL Soxhlet reagent and d ‐glucose standard. The quantity of reducing sugar was calculated using.

(3) $$\mathrm{RS}=\left[100\left(A\times B\right)\right]\;W$$

where RS = reducing sugar (in%); A = D—glucose used to reduce 20 mL soxhlet reagent (in mL) B = concentration of d‐glucose standard used to reduce 20 mL soxhlet reagent W = weight of the sample titrant used (in g).

The amylose content was determined by the ISO (2015) method. The sample (0.1 g), blank or standard was mixed with 1 mL of 95% ethanol and 9 mL of 1 M NaOH and boiled in a water bath for 20 min. This was followed by pouring 5 mL of distilled water into a 10 mL test tube and pipetting 0.5 mL of the test sample, standard or blank, 0.1 mL of 5% acetic acid, 0.2 mL of iodine and adding distilled water to make it up to the 10 mL mark. They were vortex mixed and the absorbance was read at 720 nm wavelength against the blank. A calibration curve was obtained using standard graded amylose (Fluka chemicals, Germany) and the percentage amylose was extrapolated from the curve.

### 2.5 Determination of Mineral Content

The elemental content of the samples was determined using inductively coupled plasma optical emission spectrometry (ICP‐OES) (Thermo Fisher Scientific, model iCAP 6500, Waltham, Massachusetts, USA) according to a previously described procedure (Chinma et al. 2023). The samples were digested by nitric‐hydrogen peroxide in a digiPrep hot block (SCP Science, Quebec, Canada). The rice flours (0.20 g) were digested for 45 min at 115°C. The extracts were diluted with de‐ionized water to 50 mL and left to cool to ambient temperature after which they were analyzed in the ICP‐OES equipment where the concentrations of the minerals were measured. The accuracy of the digestion was evaluated using the American National Institute of Standards and Technology, ANSI's Standard Reference Material 1573a.

### 2.6 Determination of Phytic Acid Content

Phytic acid content was measured using the method of Vaintraub and Lapteva (1988). Rice flour was extracted with 10 mL of 3.5% hydrochloric acid and stirred for 1 h using a magnetic stirrer. The mixture was centrifuged at 10,000×*g* for 10 min at 10°C, and 1 mL of the supernatant was mixed with 2 mL of 3.5% HCl and 1 mL of Wade reagent (0.03 g ferric chloride in 100 mL of 0.3% sulfosalicylic acid). The mixture was centrifuged again, and absorbance was measured at 500 nm using a Genway 6305 spectrophotometer (England).

### 2.7 Determination of Vitamin Contents

All vitamin analyses were conducted using AOAC standard methods (AOAC 2015). For Vitamin B_1_ (thiamine), 5 g of the sample was measured and homogenized in 50 mL ethanolic sodium hydroxide and then filtered through Whatman No. 1 filter paper. Furthermore, 10 mL potassium dichromate was mixed with 10 mL filtrate for color development. The absorbance of the sample was measured spectrophotometrically (Genway 6305, England) at 360 nm. The absorbance obtained from the sample extract was converted to thiamin concentration by means of a calibration curve generated using different standard concentrations of 1 to 10 μg/mL.

For vitamin B_2_ (riboflavin), a 5 g sample was extracted for 1 h with 100 mL ethyl alcohol after which the extract was filtered through Whatman No. 1 filter paper. This was followed by measuring 10 mL of the filtrate and mixing with 10 mL 5% potassium permanganate and 10 mL 3% hydrogen peroxide. The mixture was incubated in a hot water bath for 30 min, after which 2 mL of 40% sodium tetraoxosulphate (vi) was added and the volume was made up to 50 mL by addition of distilled water. It was followed by centrifuging at 1500 rpm. The concentration of vitamin B_2_ in the supernatant was determined spectrophotometrically (Genway 6305, England) at 510 nm. The absorbance obtained from the sample extract was converted to riboflavin concentration by means of a calibration curve generated using different standard concentrations (1–10 μg/mL).

For vitamin B_3_ (niacin), 5 g of the sample was treated with 50 mL of 1 N tetraoxosulphate (vi) acid for 30 min. This was followed by the addition of 0.5 mL ammonia solution and filtering through Whatman No. 1 filter paper. Subsequently, 10 mL of the filtrate was mixed with 5 mL of 0.5% potassium cyanide and was followed by acidification with 5 mL of 0.02 N tetraoxosulphate (vi) acid. The absorbance of the resulting solution was read spectrophotometrically (Genway 6305, England) at 420 nm. The absorbance obtained from the sample extract was converted to niacin concentration by means of a calibration curve generated using different standard concentrations (1–10 μg/mL).

Vitamin B_6_ (pyridoxine) was determined by weighing 1 g of the sample into a 100 mL conical flask, followed by extraction with 10 mL 0.1 M hydrochloric acid accompanied by vigorous shaking for 10 min. The sample was filtered through Whatman No. 1 filter paper and the filtrate was increased to 10 mL by the addition of distilled water. Subsequently, 5 mL of the slightly acidic filtrate was treated with 1 mL 0.40% ferric chloride. The optical density of the product which had a brown color was read in a spectrophotometer (Genway 6305, England) at 450 nm. The absorbance obtained from the sample extract was converted to pyridoxine concentration by means of a calibration curve generated using different standard concentrations (1–10 μg/mL).

Vitamin E (tocopherol) analysis was done by extracting 1 g of the sample with 50 mL petroleum ether, after which it was dried in an oven at 35°C. The residue was saponified with 5 mL of 0.1 M potassium hydroxide under reflux. Subsequently, 20 mL of petroleum ether was used to extract the unsaponified matter and the filtrate was concentrated to dryness at 35°C. Furthermore, 20 mL of ethanol was added to dissolve the concentrate, and 1 mL was transferred to a test tube, after which 1 mL of 0.2% ferric chloride in ethanol was added. Furthermore, 1 mL of 0.5% dipyridyl in ethanol (70%) was also added. Ethanol was then added to the resulting product, increasing the sample volume to 5 mL. Finally, the absorbance was read using a spectrophotometer (Genway 6305, England) at 520 nm.

### 2.8 Statistical Analysis

All analyses were conducted in triplicate. Data were subjected to Analysis of Variance (ANOVA) using the General Linear Model (GLM) procedure of SAS on Windows version 9.2. Mean comparisons were conducted using the Least Significant Difference (LSD) test, and significance was determined at *p* < 0.05.

## 3 Results and Discussion

### 3.1 Proximate Composition of GBR as Affected by Duration of Germination and Rice Cultivar

Table 1 presents the means of the proximate composition of the rice samples as affected by the rice cultivars and the durations of germination. The proximate composition of the rice samples was significantly influenced by both the cultivar type and the duration of germination. Among the cultivars, FARO 57 exhibited superior nutritional attributes, particularly in protein (13.34%), ash (2.71%), and dietary fiber (9.16%). This aligns with previous findings by Ayamdoo et al. (2013), who noted varietal differences in the nutritional composition of rice, attributing such differences to genetic variation and environmental adaptation. The elevated nutrient levels in FARO 57 suggest its potential as a functional cultivar for improving dietary quality. In contrast, FARO 44 and FARO 52 had comparable, though slightly lower, nutrient contents, particularly in protein and ash.

**TABLE 1:** Means of the rice proximate composition as affected by cultivar and durations of germination.

Treatment	Proteins (%)	Ash (%)	Total DF (%)	Fats (%)	Total CHO (%)	Moisture (%)
*Cultivars*						
FARO 44	11.58^b^ ± 1.16	1.90^b^ ±0.69	8.07^b^ ± 1.55	2.54^a^ ± 0.85	69.96^a^ ± 5.36	11.02^a^ ± 0.79
FARO 57	13.34^a^ ± 2.38	2.71^a^ ± 1.19	9.16^a^ ± 1.65	2.44^a^ ± 0.79	70.36^a^ ± 4.96	11.10^a^ ± 0.74
FARO 52	11.85^b^ ± 1.26	1.89^b^ ± 0.68	7.88^c^ ± 1.42	2.18^b^ ± 0.61	70.34^a^ ± 5.21	11.06^a^ ± 0.73
*Germination duration*						
0 h (MR)	10.16^d^ ± 0.00	1.26^c^ ± 0.00	5.20^d^ ± 0.00	1.00^c^ ± 0.00	87.15^a^ ± 0.00	10.46^c^ ± 0.00
0 h (BR)	11.01^c^ ± 0.03	1.34^c^ ± 0.03	8.18^c^ ± 0.07	2.38^b^ ± 0.06	77.47^b^ ± 0.02	12.48^a^ ± 0.02
12 h (GBR)	12.90^b^ ± 1.58	2.13^b^ ± 0.93	8.81^b^ ± 0.37	2.85^a^ ± 0.29	71.57^c^ ± 0.47	10.76^b^ ± 0.21
24 h (GBR)	13.49^ab^ ± 1.54	3.00^a^ ± 0.54	8.99^ab^ ± 0.32	2.83^a^ ± 0.26	67.33^d^ ± 1.41	10.84^b^ ± 0.12
36 h (GBR)	13.72^a^ ± 1.50	3.09^a^ ± 0.58	9.17^a^ ± 0.36	2.88^a^ ± 0.31	62.59^e^ ± 1.41	10.76^b^ ± 0.13

*Note:* Values with the same superscript in each column are not significantly different (*p* > 0.05).

Abbreviations: BR = ungerminated brown rice, CHO = carbohydrate, DF = dietary fiber, GBR = germinated brown rice, MR = ungerminated parboiled milled rice.

Germination had a profound effect on the nutritional quality of brown rice. Protein content increased significantly from 10.16% in milled rice (MR) to 13.72% after 36 h of germination, consistent with earlier reports by Kayode et al. (2020) who found that germination enhances protein synthesis through enzymatic activation and amino acid biosynthesis. This increase is also supported by Chung et al. (2009), who reported that germination stimulates protease activity, leading to the breakdown of storage proteins and the formation of new amino acids.

Ash content, which reflects mineral availability, increased progressively from 1.26% (MR) to 3.09% (36 h GBR). This agrees with the findings by Cho and Lim (2016), who observed increased mineral extractability and bioavailability in germinated grains due to phytate degradation. Similarly, the rise in dietary fiber from 5.20% to 9.17% corroborates the report of Wu et al. (2013), who found that germination activates enzymes such as cellulase and hemicellulase, contributing to higher levels of insoluble and soluble dietary fiber.

Fat content also increased with germination, from 1.00% (MR) to 2.88% (36 h GBR), a trend previously reported by Sharif et al. (2014), who associated lipid mobilization and the formation of free fatty acids with germination‐induced lipase activity. In contrast, a notable reduction in total carbohydrate content was observed as germination duration increased. Carbohydrate content decreased from 87.15% in MR to 62.59% in 36 h GBR, reflecting the hydrolysis of complex carbohydrates into simpler sugars. The sugars are subsequently utilized for sprout growth and metabolic activities supporting the findings of Tian et al. (2004), who described the hydrolysis of starch into simpler sugars utilized for sprout metabolism. This decline in carbohydrates is a typical consequence of seed respiration and enzymatic activity during germination.

The nutritional improvements observed with germination, especially up to 36 h, confirm earlier assertions that germinated brown rice (GBR) can serve as a superior dietary alternative to milled and raw brown rice (Weng et al. 2019; Ukpong et al. 2024). The FARO 57 cultivar emerged as the most promising variety, combining both high nutrient density and favorable compositional shifts during germination. These findings agree with prior studies on rice and other cereals and underscore the value of germination as a low‐cost, natural technique to improve the functional and nutritional quality of rice products (Patil and Khan 2011; Kayode et al. 2020). This supports its potential use in the development of value‐added rice products aimed at improving public nutrition and health outcomes.

### 3.2 Effect of Cultivar and Duration of Germination on the Functional Properties of GBR

The functional properties of rice samples, including bulk density (BD), foaming capacity (FC), foaming stability (FS), water absorption capacity (WAC), oil absorption capacity (OAC), and swelling power (SP), were significantly affected (*p* < 0.05) by both rice cultivar and germination duration (Table 2).

**TABLE 2:** Means of the rice functional properties as affected by cultivar and durations of germination.

Treatment	BD (g/mL)	FC (%)	FS (%)	WAC (g/g)	OAC (g/g)	SP (g/g)
*Cultivar*						
FARO 44	0.80^c^ ± 0.15	62.91^b^ ± 8.41	56.90^b^ ± 13.01	1.99^c^ ± 0.68	1.91^b^ ± 0.80	4.90^b^ ± 1.75
FARO 57	0.85^b^ ± 0.11	67.23^a^ ± 11.12	60.45^a^ ± 9.67	2.36^a^ ± 0.56	2.33^a^ ± 0.49	5.09^a^ ± 1.61
FARO 52	0.90^a^ ± 0.09	62.56^b^ ± 7.84	58.62^ab^ ± 8.85	2.16^b^ ± 0.63	2.25^a^ ± 0.48	5.09^a^ ± 1.60
*Germination duration*						
0 h (MR)	0.97^a^ ± 0.01	51.13^d^ ± 0.07	44.37^e^ ± 2.14	1.44^e^ ± 0.22	1.48^e^ ± 0.32	6.86^a^ ± 0.01
0 h (BR)	0.96^a^ ± 0.02	57.31^c^ ± 2.17	51.16^d^ ± 6.04	1.53^d^ ± 0.24	1.66^d^ ± 0.44	6.80^a^ ± 0.02
12 h (GBR)	0.87^b^ ± 0.06	66.75^b^ ± 0.26	60.85^c^ ± 0.30	2.25^c^ ± 0.12	2.13^c^ ± 0.13	4.80^b^ ± 0.10
24 h (GBR)	0.76^c^ ± 0.10	72.24^a^ ± 4.74	64.01^b^ ± 0.70	2.69^b^ ± 0.17	2.54^b^ ± 0.11	3.81^c^ ± 0.25
36 h (GBR)	0.70^d^ ± 0.07	73.71^a^ ± 4.30	72.88^a^ ± 3.01	2.93^a^ ± 0.09	3.01^a^ ± 0.08	2.86^d^ ± 0.13

*Note:* Values with the same superscript in each column are not significantly different (*p* > 0.05).

Abbreviations: BD = bulk density, BR = ungerminated brown rice, FC = foaming capacity, FS = foam stability, GBR = germinated brown rice, MR = ungerminated parboiled milled rice, OAC = oil absorption capacity, SP = swelling power, WAC = water absorption capacity.

FARO 52 had the highest BD (0.90 g/mL), followed by FARO 57 (0.85 g/mL), while FARO 44 had the lowest (0.80 g/mL). A higher BD indicates a denser flour, which is essential in packaging and transport (Otegbayo et al. 2014). The variation among cultivars could be attributed to inherent differences in grain structure and composition. FARO 57 also exhibited significantly higher FC (67.23%) and FS (60.45%) compared to the other cultivars. Proteins and saponins are largely responsible for foaming properties; thus, the observed difference may relate to protein quality and surface‐active components in FARO 57 (Adebowale et al. 2005). FARO 57 also showed the highest WAC (2.36 g/g) and OAC (2.33 g/g), suggesting a higher ability to retain moisture and oil, which enhances the mouthfeel and flavor retention in food products. These differences are cultivar‐dependent and relate to starch granule structure and hydrophilic group interactions (Kaur et al. 2012). FARO 52 and FARO 57 had the highest SP (5.09 g/g), which is useful in food formulations requiring high viscosity. This could be attributed to varietal differences in starch composition, particularly amylopectin levels (Zhu 2015) as shown in Table 3.

**TABLE 3:** Means of the total starch, amylose and total reducing sugars content of rice as affected by cultivar.

Treatment	Total starch (%)	Amylose (%)	Total reducing sugars (%)
*Cultivar*			
FARO 44	63.74^a^ ± 7.36	29.43^a^ ± 5.56	5.49^a^ ± 4.56
FARO 57	63.31^a^ ± 7.74	30.11^a^ ± 5.08	6.04^a^ ± 4.18
FARO 52	63.61^a^ ± 7.64	29.61^a^ ± 5.41	6.24^a^ ± 4.39

*Note:* Values are means and SDs and values with the same superscript in each column are not significantly different (*p* > 0.05).

There was a consistent decline in BD with germination—from 0.97 g/mL (MR) to 0.70 g/mL (36 h GBR). Germination softens the grain matrix and increases porosity due to enzymatic breakdown of macromolecules (Amadou et al. 2011). FC and FS significantly increased with germination duration, peaking at 36 h (73.71% and 72.88%, respectively). This suggests protein denaturation and unfolding during germination, which exposes hydrophobic groups that stabilize foam (Afify et al. 2012). WAC significantly increased from 1.44 g/g (MR) to 2.93 g/g (36 h GBR), indicating improved hydration potential of GBR. This increase is consistent with partial hydrolysis of starch and protein matrices, allowing more water‐binding sites (Tiansawang et al. 2016). OAC also increased significantly with germination, from 1.48 g/g (MR) to 3.01 g/g (36 h GBR). This is potentially beneficial in formulating meat analogues and baked goods, as higher oil‐binding enhances palatability (El‐Adawy 2002). SP decreased significantly from 6.86 g/g (MR) to 2.86 g/g (36 h GBR). The reduction may be linked to enzymatic degradation of starch granules during sprouting, reducing their capacity to swell (Saleh et al. 2013).

### 3.3 Total Starch, Amylose and Total Reducing Sugar Contents

Table 3 presents the means of the total starch, amylose and total reducing sugar composition of the samples as affected by the rice cultivars. The total starch composition as affected by the rice cultivars were in the range of 63.31%–63.74% and no significant differences (*p* > 0.05) existed among the three rice cultivars. The means of the amylose content ranged from 29.43%–30.11% and there was also no significant difference (*p* > 0.05) among the three rice cultivars. Furthermore, the total reducing sugars ranged from 5.49%–6.24% and there were also no significant differences (*p* > 0.05) among the three rice cultivars.

**FIGURE 1:**
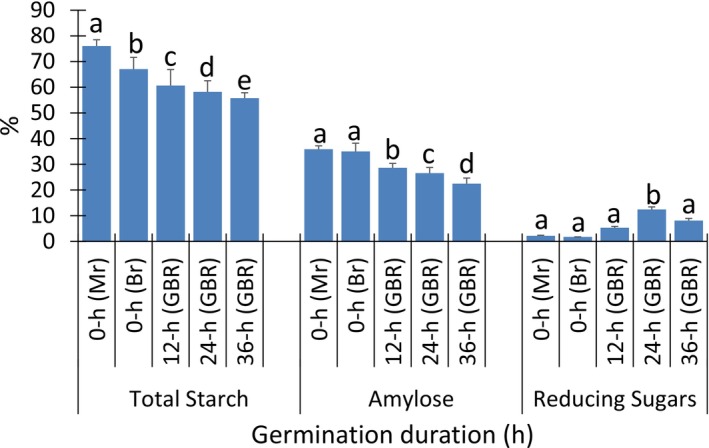
Means of the total starch, amylose and total reducing sugars compositions as affected by germination duration. Values are mean ± SD of triplicate determinations. Values with the same superscript for each carbohydrate type are not significantly different (*p* > 0.05). BR = Ungerminated brown rice, GBR = Germinated brown rice, MR = Ungerminated parboiled milled rice.

Figure 1 shows the means of the total starch, amylose and total reducing sugars compositions as affected by the duration of germination. The average of the total starch was 76.06% in MR, and reduced to 67.07% in BR and was further reduced to the range of 55.76%–60.67% in GBR samples.

### 3.4 Macro Mineral Composition of Germinated Brown Rice

Table 4 presents the macro mineral content of the GBR samples. Across all cultivars, the contents of calcium, potassium, magnesium, phosphorus, sodium, and sulfur significantly increased following germination compared to the non‐germinated control (*p* < 0.05). This finding aligns with previous studies indicating that germination enhances nutrient extractability by modifying grain structure (Benincasa et al. 2019; Weng et al. 2019; Ukpong et al. 2024). For FARO 44, the optimal germination duration for mineral accumulation was 24 h, resulting in peak concentrations of calcium (123.33 mg/100 g), magnesium (1299.24 mg/100 g), and phosphorus (4233.42 mg/100 g). A subsequent decline at 36 h may be attributed to nutrient utilization by growing shoots (Chaijan and Panpipat 2020). Similar trends were observed in FARO 52 and FARO 57, although mineral accumulation patterns varied slightly by cultivar. This indicates that both germination time and variety influence nutrient dynamics, consistent with Kaur et al. (2017) and Loan and Thuy (2020). Importantly, the improved mineral profiles suggest GBR could serve as a dietary intervention in nutrient‐deficient populations. For instance, 16.54 g/day of FARO 44 GBR germinated for 24 h provides the RDA of phosphorus (700 mg/day) for adults (FNB 2001). Likewise, magnesium RDAs could be met by 32.33 g/day (males) or 24.63 g/day (females) of the same sample.

**TABLE 4:** Macro mineral composition (mg/100 g) of parboiled GBR as affected by germination duration.

Cultivar	Germination time (h)	Ca (mg/100 g)	K (mg/100 g)	Mg (mg/100 g)	Na (mg/100 g)	P (mg/100 g)	S (mg/100 g)
FARO 44	0	111.07 ± 1.04^e^	2508.38 ± 2.45^b^	1064.26 ± 2.42^f^	20.73 ± 0.53^b^	3901.67 ± 3.04^d^	1108.68 ± 2.87^d^
FARO 44	12	114.78 ± 1.23^e^	2397.25 ± 3.01e	1181.90 ± 2.70^c^	23.82 ± 0.43^a^	3995.27 ± 3.56^b^	1123.67 ± 2.50^c^
FARO 44	24	123.33 ± 1.27^c^	2444.39 ± 2.11^cd^	1299.24 ± 2.22^a^	17.06 ± 0.79^c^	4233.42 ± 2.97^a^	1202.37 ± 2.87^a^
FARO 44	36	115.31 ± 1.30^e^	2106.91 ± 3.45^g^	1119.33 ± 2.96^e^	14.53 ± 0.81^d^	3694.09 ± 3.44^f^	1087.42 ± 2.59^e^
FARO 52	0	86.09 ± 1.02^g^	2104.99 ± 3.18^g^	1066.67 ± 3.01^f^	7.67 ± 0.54^g^	3240.70 ± 3.51^j^	901.41 ± 3.01^h^
FARO 52	12	103.11 ± 1.28^f^	1980.75 ± 3.11^h^	1188.66 ± 1.97^c^	11.88 ± 0.61^e^	3632.99 ± 3.71^g^	958.17 ± 2.67^g^
FARO 52	24	180.62 ± 0.98^a^	1939.98 ± 3.62^i^	1158.67 ± 2.12^d^	9.44 ± 0.73^f^	3471.76 ± 3.11^hi^	965.03 ± 2.74^g^
FARO 52	36	115.53 ± 1.11^de^	1781.24 ± 3.43^j^	1141.40 ± 2.74^d^	9.48 ± 0.65^f^	3468.14 ± 3.67^i^	866.53 ± 2.69^i^
FARO 57	0	85.27 ± 1.21^g^	2538.51 ± 3.15^a^	1193.04 ± 2.11^bc^	2.37 ± 0.38^i^	3678.90 ± 2.58^f^	1015.57 ± 2.85^f^
FARO 57	12	119.20 ± 1.01^cd^	2429.95 ± 2.94^d^	1220.51 ± 2.43^b^	5.33 ± 0.61^h^	3954.41 ± 3.00^c^	1162.88 ± 2.49^b^
FARO 57	24	120.71 ± 1.31^c^	2457.27 ± 3.07^c^	1209.98 ± 2.32^b^	5.91 ± 0.56^h^	3774.95 ± 2.97^e^	1153.65 ± 2.56^b^
FARO 57	36	130.03 ± 1.19^b^	2304.82 ± 3.19^f^	1224.40 ± 2.71^b^	5.74 ± 0.81^h^	3493.67 ± 2.74^h^	1150.33 ± 2.81^b^

*Note:* Values are mean ± SD of triplicate determinations. Values in each column with the same superscript are not significantly different at *p* > 0.05.

### 3.5 Trace Mineral Composition of Germinated Brown Rice

Table 5 shows the trace mineral content of the brown rice as affected by the duration of germination. The trace minerals (mg/100 g) were in the following ranges: 17.21 to 87.69 for iron, 16.18 to 25.51 for zinc, 1.24 to 2.64 for copper, 22.99 to 40.68 for manganese, 0.70 to 2.02 for selenium, 0.03 to 0.80 for nickel, 1.99 to 2.79 for cerium, and 52.67 to 96.84 for silicon. Like the macro minerals, significantly (*p* > 0.05) higher trace micro minerals were observed among the germinated samples compared to the non‐germinated samples, which also agrees with previous studies (Chinma et al. 2015; Sathe et al. 2020). Significant differences were also observed among the GBR samples at different germination times. Data from the trace mineral contents of FARO 44, FARO 52, and FARO 57cultivars showed changes that were germination time dependent. An increase was observed, from 12 to 24 h germination, whereas germination to 36 h resulted in a decrease in the contents of these minerals. The decrease at the germination time of 36 h could be attributed to the utilization of these minerals by the shoots. Exceptions to this were found in nickel where a significant (*p* > 0.05) increase was observed in FARO 44 and FARO 52 at 24 h, while no significant effect occurred by increasing the time to 36 h. Similarly, the cerium content showed no significant difference in FARO 44 and FARO 52, when the germination time was increased to 24 h while a further increase to 36 h resulted in significantly higher cerium content.

**TABLE 5:** Trace mineral composition of parboiled GBR as affected by germination duration.

Cultivar	Germination time (h)	Fe (mg/100 g)	Zn (mg/100 g)	Cu (mg/100 g)	Mn (mg/100 g)	Se (mg/100 g)	Ni (mg/100 g)	Ce (mg/100 g)	Si (mg/100 g)
FARO 44	0	35.43 ± 0.26^d^	20.66 ± 0.18^c^	1.24 ± 0.05^h^	22.99 ± 0.16^e^	1.46 ± 0.04^c^	0.03 ± 0.00^f^	1.99 ± 0.02^g^	72.04 ± 1.47^d^
FARO 44	12	66.01 ± 0.29^c^	23.94 ± 0.21^b^	2.16 ± 0.05^e^	26.40 ± 0.20^d^	2.02 ± 0.03^a^	0.07 ± 0.00^f^	2.16 ± 0.01^e^	74.66 ± 1.60^c^
FARO 44	24	87.69 ± 0.24^a^	25.51 ± 0.17^a^	1.77 ± 0.07^f^	40.68 ± 0.21^a^	1.97 ± 0.05^a^	0.09 ± 0.00^f^	2.41 ± 0.03^c^	97.40 ± 1.35^a^
FARO 44	36	70.36 ± 0.23^b^	20.39 ± 0.23^c^	1.60 ± 0.04^g^	34.97 ± 0.19^b^	1.67 ± 0.03^b^	0.44 ± 0.02^d^	2.49 ± 0.03^c^	74.62 ± 1.52^c^
FARO 52	0	18.12 ± 0.19^hi^	16.18 ± 0.26^d^	2.35 ± 0.05^c^	24.03 ± 0.21^e^	0.93 ± 0.02^e^	0.56 ± 0.02^c^	2.23 ± 0.01^d^	52.67 ± 1.34^g^
FARO 52	12	17.21 ± 0.27^i^	17.08 ± 0.21^d^	2.42 ± 0.07^b^	27.99 ± 0.18^d^	1.14 ± 0.04^d^	0.55 ± 0.01^c^	2.26 ± 0.03^d^	66.04 ± 1.25^e^
FARO 52	24	19.46 ± 0.27^h^	19.30 ± 0.21^c^	2.64 ± 0.08^a^	30.34 ± 0.15^c^	1.71 ± 0.05^b^	0.65 ± 0.03^b^	2.57 ± 0.02^b^	71.02 ± 1.47^d^
FARO 52	36	30.15 ± 0.19^e^	17.66 ± 0.19^d^	2.48 ± 0.05^b^	27.23 ± 0.21^d^	0.70 ± 0.01^f^	0.80 ± 0.01^a^	2.59 ± 0.02^b^	65.67 ± 1.53^e^
FARO 57	0	22.14 ± 0.20^g^	19.79 ± 0.24^c^	1.72 ± 0.05^f^	23.68 ± 0.21^e^	1.44 ± 0.01^c^	0.22 ± 0.01^e^	2.08 ± 0.01^f^	57.58 ± 1.54^f^
FARO 57	12	28.27 ± 0.28^f^	24.30 ± 0.21^ab^	2.08 ± 0.05^e^	30.00 ± 0.17^c^	1.69 ± 0.04^b^	0.23 ± 0.02^e^	2.28 ± 0.04^d^	96.38 ± 1.67^a^
FARO 57	24	31.86 ± 0.25^e^	23.40 ± 0.20^b^	2.29 ± 0.07^d^	35.78 ± 0.19^b^	1.93 ± 0.03^a^	0.39 ± 0.02^d^	2.60 ± 0.02^b^	96.84 ± 1.23^a^
FARO 57	36	18.04 ± 0.21^hi^	23.05 ± 0.25^b^	2.25 ± 0.05^d^	34.23 ± 0.17^b^	2.02 ± 0.04^a^	0.43 ± 0.02^d^	2.79 ± 0.03^a^	94.57 ± 1.67^b^

*Note:* Values are mean ± SD of triplicate determinations. Values in each column with the same superscript are not significantly different at *p* > 0.05.

Similar to the macro minerals, the RDA of the trace minerals could easily be attained by consuming the parboiled GBR instead of the non‐GBR. For example, consumption of 9.12 and 20.52 g/day of GBR from FARO 44 germinated for 24 h by males and females, respectively, could meet the RDA of 8 and 18 g/day of iron for males (19 and above) and females (19–50 years), respectively (FNB 2001). Also, consumption of 43.12 and 31.36 g/day of FARO 44 germinated for 24 h by males and females could meet the RDA of zinc, which is 11 and 8 g/day for males (14 years and above) and females (19 years and above) (FNB 2001; Ren et al. 2020).

It is also worth stressing the selenium and cerium contents of the GBR due to their unconventional, yet important functions. As reported earlier, selenium and cerium contents were much higher in the parboiled GBR, compared to the control. Selenium, a well‐known antioxidant, helps to boost immunity, defend the body against oxidative stress and protect the heart (Tsuji et al. 2016). Its deficiency is reported to be responsible for Keshan disease, characterized by pulmonary edema and heart failure; Kashin–Beck disease which causes inflammation of bones and joint necrosis; and myxedematous endemic cretinism, which is characterized by mental retardation (Kang et al. 2020). On the other hand, the physiological role of cerium, a rare earth element, is not fully understood. However, there are reports that it helps to prevent neurodegenerative diseases, inflammation, facilitate wound healing, serve as an antioxidant by helping to reduce oxidative stress, as well as its anti‐aging effects (Yi et al. 2024; Kang et al. 2020).

Contrary to our findings, lower zinc (1.79–1.90 mg/100 g) and iron (2.85–3.97 mg/100 g) contents in GBR produced by germinating other local cultivars of paddy rice (*Jamila*, *Jeep* and *Kwandala*) *were* reported (Chinma et al. 2015). The differences could be ascribed to differences in the rice cultivars, agro ecological zones, and types of fertilizer used to produce the rice (Cho and Lim 2016). The values we obtained in the present study are also higher than the ranges for selenium (80.10–107.50 μg/100 g), zinc (1.68–2.72 mg/100 g) and iron (3.15–9.94 mg/100 g) previously reported when dehusked rice from these cultivars was germinated (Ukpong et al. 2024).

### 3.6 Vitamin Composition of Germinated Brown Rice

Table 6 shows the vitamin contents of the samples. Vitamin B_1_ (mg/100 g) ranged from 0.15 mg in FARO 57 germinated for 0 h to 0.78 mg in FARO 44 germinated for 36 h. Vitamin B_2_ (mg/100 g) ranged from 0.05 mg in FARO 44 germinated for 0 h to 0.65 mg in FARO 57 germinated for 36 h. Vitamin B_3_ (mg/100 g) ranged from 1.09 mg in FARO 52 germinated for 0 h to1.51 mg in FARO 44 germinated for 36 h. Vitamin B_6_ content (mg/100 g) ranged from 5.48 mg in FARO 57 at 0 h germination to 9.47 mg in FARO 57 at 36 h germination. Vitamin E content (mg/100 g) ranged from 1.90 mg in FARO 52 at 0 h germination to 3.02 mg in FARO 57 at 36 h germination. Notable significant differences (*p* > 0.05) were observed in the contents of the vitamins between the controls and the germinated samples. This suggests that the germination process strongly affected the vitamin contents, which agrees with previous results (Chaijan and Panpipat 2020; Ukpong et al. 2023). This finding also suggested the possibility of de novo bio‐synthesis of these vitamins in the course of the germination process. Notably, the vitamins increased significantly as the duration of germination increased, except in FARO 52 where no significant (*p* > 0.05) differences were observed between the samples germinated for 24 and 36 h. At the germination duration of 36 h, vitamin B_1_ increased by 388%, 285%, and 300%; vitamin B_2_ increased by 440%, 358%, and 110%; vitamin B_3_ increased by 23%, 36%, and 9%; vitamin B_6_ increased by 31%, 42%, and 72%; and vitamin E increased by 15%, 57%, and 53%, for FARO 44, FARO 52 and FARO 57, respectively. The possible explanation for the increase in the vitamin contents as a function of germination time could be due to an increase in enzymatic activity (Ren et al. 2020). Thus, for improved vitamin content, the paddy should be germinated for at least 36 h to facilitate attaining their Recommended Dietary Allowances (RDA). For example, with FARO 44 and FARO 52, consumption of 153.85 g/day and 141.03 g/day of GBR germinated for 36 h by males and females, respectively, could meet the RDA of thiamin which is 1.2 mg/day and 1.1 mg/day for males (14 years and above) and females (19 years and above) respectively (FNB 2001; 2011). Furthermore, consumption of 216.67 g/day GBR of FARO 57, corresponding to the paddy germination for 36 h could meet the RDA of riboflavin, which is 1.3 mg/day for males (14 years and above), while 183.33 g/day of the GBR could meet the RDA of 1.1 mg/day for females (19 years and above) (FNB 2001). For vitamin B_6_, the RDA of 1.3 mg/day for males (14–50 years) and females (19–50 years) could be achieved by consuming 13.73 g/day of the GBR of FARO 57 that was germinated for 36 h (FNB 2001).

**TABLE 6:** Vitamin composition (mg/100 g) of parboiled GBR as affected by germination duration.

Sample	Vitamin B_1_	Vitamin B_2_	Vitamin B_3_	Vitamin B_6_	Vitamin E
F44‐0 h	0.16 ± 0.01^f^	0.05 ± 0.01^g^	1.23 ± 0.01^e^	6.79 ± 0.19^f^	2.57 ± 0.05^ef^
F44‐12 h	0.42 ± 0.02^d^	0.16 ± 0.01^f^	1.33 ± 0.01^cd^	7.94 ± 0.15^d^	2.76 ± 0.07^cd^
F44‐24 h	0.65 ± 0.02^b^	0.23 ± 0.01^e^	1.43 ± 0.02^b^	8.41 ± 0.17^c^	2.89 ± 0.07^b^
F44‐36 h	0.78 ± 0.03^a^	0.27 ± 0.02^de^	1.51 ± 0.02^a^	8.89 ± 0.19^b^	2.96 ± 0.05^ab^
F52‐0 h	0.20 ± 0.01^e^	0.12 ± 0.01^f^	1.09 ± 0.01^g^	5.62 ± 0.12^h^	1.90 ± 0.08^g^
F52‐12 h	0.53 ± 0.03^c^	0.37 ± 0.03^d^	1.20 ± 0.02^e^	6.84 ± 0.17^f^	2.49 ± 0.09^ef^
F52‐24 h	0.77 ± 0.03^a^	0.53 ± 0.02^b^	1.32 ± 0.02^cd^	7.57 ± 0.14^e^	2.68 ± 0.04^d^
F52‐36 h	0.77 ± 0.02^a^	0.55 ± 0.02^b^	1.37 ± 0.03^bc^	7.99 ± 0.15^d^	2.98 ± 0.05^a^
F57‐0 h	0.15 ± 0.01^f^	0.31 ± 0.03^d^	1.16 ± 0.01^f^	5.48 ± 0.18^h^	2.00 ± 0.03^g^
F57‐12 h	0.21 ± 0.02^e^	0.45 ± 0.02^c^	1.23 ± 0.02^e^	6.21 ± 0.14^g^	2.45 ± 0.06^f^
F57‐24 h	0.39 ± 0.03^d^	0.52 ± 0.02^b^	1.32 ± 0.03^cd^	7.54 ± 0.15^e^	2.49 ± 0.03^ef^
F57‐36 h	0.60 ± 0.03^bc^	0.65 ± 0.02^a^	1.27 ± 0.02^de^	9.47 ± 0.18^a^	3.02 ± 0.05^a^

*Note:* Values are mean ± SD of triplicate determinations. Values in each column with the same superscript are not significantly different at *p* > 0.05. F = FARO; 44, 52 and 57 are different FARO cultivars; 0, 12, 24 and 36 are germination durations.

A comparison of the vitamin contents obtained in the present study to data obtained when the dehusked grains were germinated (Maksup et al. 2021) showed that vitamin contents in the current study were higher than the reported ranges of 0.33 to 0.35 mg/100 g for vitamin B_1_; 0.82 to 0.96 mg/100 g for vitamin B_3_; and 0.90 to 1.17 mg/100 g for vitamin B_6_ in the dehusked grains, but were similar to the ranges of 1.17 to 2.87 mg/100 g reported for vitamin E. It is most likely that there was a higher rate of leaching of the B‐group vitamins as a result of the repeated sprinkling of water when the dehusked grains were germinated, compared to when the paddy was germinated, resulting in the observed disparity.

### 3.7 Phytic Acid Composition of Germinated Brown Rice

**FIGURE 2:**
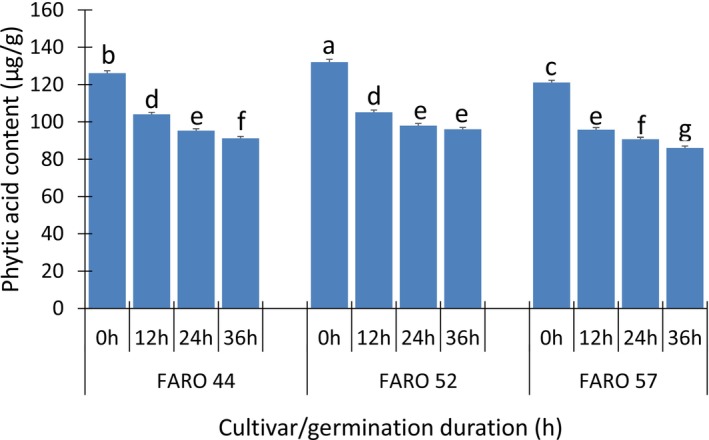
Phytic acid composition (μg/g) of the parboiled GBR as affected by the duration of germination. Values are mean ± SD of triplicate determinations. Values in each column with the same superscript are not significantly different across cultivars and germination time (*p* > 0.05). 0 h, 12 h, 24 h, and 36 h are germination durations.

The phytic acid contents of the rice samples ranged from 83.03 to 132.04 μg/g (Figure 2). The phytic acid contents of the germinated samples were all significantly (*p* > 0.05) lower than those of the non‐germinated samples. Reduction in phytic acid in the germinated samples could be attributed to the activity of the enzyme, *phytase*, during the germination of the paddy rice. The phytic acid contents were further significantly (*p* > 0.05) reduced as the duration of germination increased, which could be attributed to an increase in *phytase* activity (Kaur et al. 2017). Phytic acid is a mineral inhibitor that chelates cations such as calcium, phosphorus, magnesium, zinc, and iron to form a complex that renders such minerals bio‐unavailable. Thus, GBR does not only have higher mineral content than the non‐germinated samples but also has lower phytic acid content, which suggests increased bioavailability of these minerals.

## 4 Conclusion

This study demonstrated that controlled germination of paddy rice, before standard parboiling and milling, significantly improves the nutritional quality of GBR. Across all tested cultivars (FARO 44, FARO 52, and FARO 57), germination enhanced the contents of essential macro‐ and trace minerals, including calcium, magnesium, phosphorus, iron, zinc, and selenium, while decreasing phytic acid levels, indicating improved mineral bioavailability. The vitamin (B1, B2, B3, B6, and E) concentrations also increased substantially, particularly after 36 h of germination. These improvements have implications for food and nutrition security in regions where rice is a staple, but nutritional deficiencies are prevalent. By aligning GBR production with existing rice processing methods, this approach requires minimal infrastructural change, making it suitable for integration into local and industrial rice value chains.

The study also demonstrated that both rice cultivar and duration of germination significantly impacted the functional properties of rice flour. Notably, germination enhanced hydration‐related properties (WAC, OAC, FC), though it reduces bulk density and swelling power. FARO 57 appears to exhibit superior functional performance, making it a potentially valuable cultivar for functional food development. Furthermore, the study confirms that both germination duration and cultivar selection critically influence nutrient composition. FARO 44 germinated for 24–36 h emerged as a nutritionally optimal choice. These findings support the use of GBR as a functional food for managing metabolic disorders such as anemia, hypertension, and diabetes (Weng et al. 2019; Zhao et al. 2018).

Future research is envisaged that focus on the integration of GBR into national public health nutrition strategies and school feeding programs, which could offer a sustainable solution to combating hidden hunger in rice‐dependent populations. In addition, animal trials are strongly recommended to relate the findings of the compositional profile to real‐time health status of consumers. This would help to demonstrate how the nutritional components of the GABA rice influence the prevention and management of metabolic disorders such as obesity, type 2 diabetes, and cardiovascular diseases. Such investigations would provide translational evidence that goes beyond compositional analysis, offering insights into the bioavailability, metabolic pathways, and long‐term health impacts of GABA rice. Flour from the GABA rice described in this study can be further investigated for their applicability for other value‐added products such as beverage and pasta as already demonstrated (Ha et al. 2022).

## Author Contributions

**Uloma E. Onyeka:** conceptualization (lead), formal analysis (lead), funding acquisition (lead), investigation (lead), methodology (lead), project administration (lead), supervision (lead), writing – original draft (equal), writing – review and editing (supporting). **Ekpeno Sunday Ukpong:** conceptualization (equal), investigation (supporting), methodology (supporting), software (supporting), validation (supporting), writing – original draft (equal), writing – review and editing (supporting). **Chinedu B. Azudialu:** conceptualization (supporting), investigation (supporting), methodology (supporting), visualization (supporting). **Sali A. Ndindeng:** data curation (supporting), funding acquisition (supporting), methodology (supporting), resources (supporting), validation (supporting). **Kalimuthu Senthilkumar:** data curation (supporting), funding acquisition (supporting), methodology (supporting), resources (supporting), validation (supporting). **Christian O. Dimkpa:** data curation (supporting), formal analysis (equal), resources (supporting), software (supporting), validation (supporting), writing – review and editing (lead).

## Funding

This work was supported by the Tertiary Education Trust Fund (TETfund) of Nigeria and the Department of Food Science and Technology, School of Engineering and Engineering Technology, Federal University of Technology, Owerri, Imo State, Nigeria. Additional funding was gratefully provided by the European Union. The project 101083388: Combating malnutrition in Africa through diversification of the food system (HealthyDiets4Africa). The views and opinions expressed are, however, those of the author(s) only and do not necessarily reflect those of the European Union or the European Research Executive Agency. Neither the European Union nor the granting authority can be held responsible for them.

## Conflicts of Interest

The authors declare no conflicts of interest.

## Data Availability

Data Availability Statement: The data that support the findings of this study are available upon request from the corresponding author.
